# Breast cancer in lactating mothers: A case series of delayed diagnosis

**DOI:** 10.1016/j.ijscr.2022.107856

**Published:** 2022-12-31

**Authors:** Kanchana Wijesinghe, Umesh Jayarajah, Hasangi Gamage, Sumali De Silva, Ajith De Silva

**Affiliations:** a University Surgical Unit, Faculty of Medical Sciences, University of Sri Jayewardenepura, Sri Lanka; b Department of Surgery, National Hospital of Sri Lanka, Colombo, Sri Lanka

**Keywords:** Breast cancer, Breastfeeding, Lactation, Case series, Sri Lanka

## Abstract

**Introduction and importance:**

Due to pregnancy related changes in the breast, a breast feeding mother as well as the health care professionals may attribute a change in the breast for lactation resulting in a delay in evaluation and diagnosis. We report a cases series of delayed diagnosis of breast cancer during lactation in three young patients from a developing country who had sought medical attention on time but was unfortunately diagnosed late.

**Presentation of case:**

We report three breast feeding mothers aged 38–39 years presenting with breast lump and edema. All patients had an ultrasonography of breast performed at least once and the radiological findings were attributed to physiological changes initially despite non-resolving symptoms for 3–4 months. Triple assessment revealed invasive breast cancers of T4N1M0, T2N1M0, T3N2M1 staging. Two patients were treated with a curative intent and the patient with metastatic cancer was referred for palliative chemotherapy.

**Clinical discussion:**

Lactational mastitis, breast abscesses, galactoceles, breast edema are benign conditions that are unique in lactation period, but it is important not to overlook that the lactating women may develop any of the other breast problems seen in the non-lactating female population.

**Conclusion:**

Our case series represent very similar scenarios of delayed or missed diagnosis of breast cancer in young lactating women. A lactating patient should be referred to a specialist center and/or a complete assessment of the breast should be performed in case of any red flags findings to avoid missing a sinister diagnosis.

## Highlights

• Breast cancer in young lactating women may be overlooked resulting in a diagnostic delay.
• Clinical and radiological assessment of a breast lump becomes quite challenging in the lactating breast.
• Atypical findings during lactation should prompt a complete assessment including histology.

## 1 Introduction

Breast cancer is the most common malignancy among reproductive-aged women in both the developing and developed world [1]. Around 5 %–7 % of breast cancer cases occur in women under 40 years of age [2]. Among the many risk factors for the development of breast cancer, breastfeeding is a well-known protective factor. However, this effect is not immediate or constant. Development of breast cancer during pregnancy and lactation is rare and the incidence is approximately 1 in 3000 [3]. The prevalence of pregnancy associated breast cancer is noted to be increasing owing to the delayed child bearing. Despite its low incidence, breast cancer is one of the commonest cancers among pregnant and lactating women [3].

Pregnancy and lactation cause many physiological changes in the breasts. These unique physiologic changes occur due to hormonal changes resulting in increased breast volume with associated nodularity, firmness and increased parenchymal density [4]. These changes progress as pregnancy advances and are maintained during lactation and will steadily change back to the pre-pregnancy state approximately 3 months after cessation of breastfeeding. Thus, during the pregnancy and lactation, the clinical and radiological evaluation of the breast becomes difficult and misleading [4].

Due to this pregnancy related changes in the breast, a breast feeding mother as well as the health care professionals may attribute a change in the breast for lactation resulting in a delay in evaluation and diagnosis. This false assurance may put a breastfeeding mother at risk of progressing into advanced breast cancer and may hinder the possibility for a cure. Breast cancer in younger women tends to be more aggressive and the tumors tend to be higher in grade, hormone receptor negative, have increased HER2/Neu overexpression, and more lympho-vascular invasion [2]. Thus early diagnosis can improve the outcome significantly [2]. Current evidence suggests that in women aged <45 years, breast cancer is the leading cause of cancer-related deaths [5].

Breast cancer is the commonest overall cancer in Sri Lanka with significant impact on quality of life [6], [7]. Furthermore, breast cancer incidence is increasing rapidly in Sri Lanka despite the absence of nationwide mammography screening policies [8]. Around two thirds of the breast cancers in Sri Lanka are diagnosed at an early stage (stage I & II), which is much lower than the developed world [9]. It is in this background that we report a cases series of delayed diagnosis of breast cancer during lactation in three young patients from a developing country who had sought medical attention on time but was unfortunately diagnosed late. The work has been reported based on the SCARE 2020 criteria [10].

## 2 Presentation of case

### 2.1 Patient 1

**Fig. 1:**
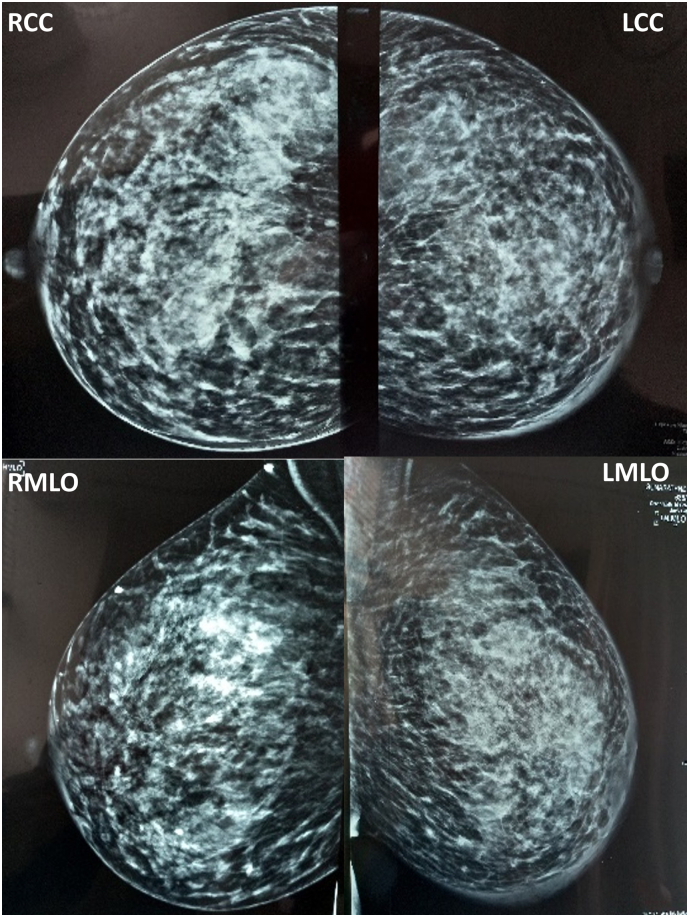
Patient 1 - Mammogram showing skin thickening and trabecular thickening of the left breast with focal asymmetry in the left sub areolar region. (RCC- Right cephalocaudal, LCC- Left cephalocaudal, RMLO- Right mediolateral oblique, LMLO- Left mediolateral oblique).

**Fig. 2:**
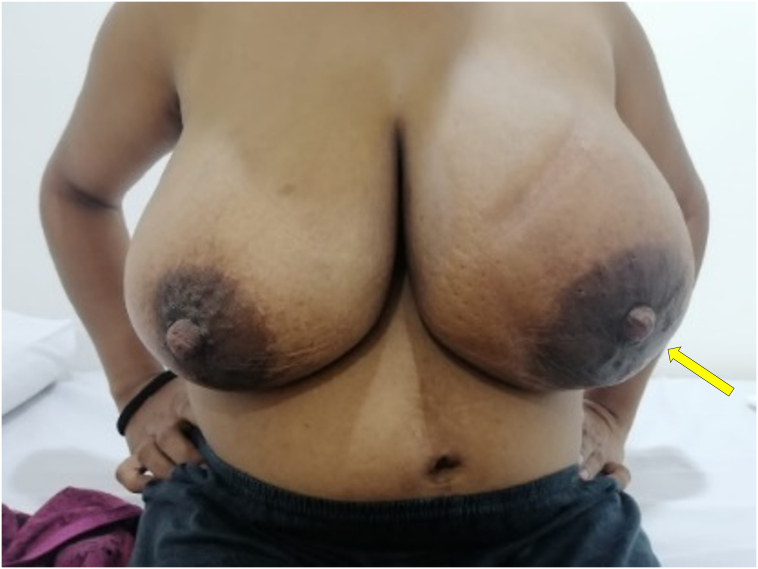
Patient 1 - Image at the time of presentation to the breast clinic showing left breast fullness and peau d'orange appearance.

A 38-year-old multiparous mother presented with a left breast lump which she had noticed immediately following birth of her third child. She was seen by the medical team on several occasions including a surgeon and a radiologist and was reassured and treated as for galactocele with lactational mastitis. The ultrasound scan of the breast was reported as a mixed echogenic mass suggestive of a galactocele and the BIRADS score was not mentioned. She presented to the breast clinic at 4 months as it was not resolving. Clinical assessment showed an asymmetrical enlargement of the left breast with peau d'orange appearance of the skin. She had no family history of breast cancer and had an uneventful past medical, surgical and allergy history. The mammogram showed skin thickening and trabecular thickening of the left breast with focal asymmetry in the left sub areolar region. Ultrasonography revealed a heterogeneous hypoechoic lesion with irregular margins measuring 24 mm × 22 mm suspicious of a malignancy. Multiple enlarged lymph nodes were seen with loss of fatty hilum, largest measuring 20 mm in diameter (BIRADS IVC). Following work up, she was diagnosed with a locally advanced stage III invasive breast cancer, T4N1M0. Her hormonal subtype was ER/PR negative, HER2 positive and Ki 67–15–20 %. She is currently undergoing neo-adjuvant chemotherapy and is planned for mastectomy with reconstruction and axillary node clearance (Fig. 1, Fig. 2).

### 2.2 Patient 2

**Fig. 3:**
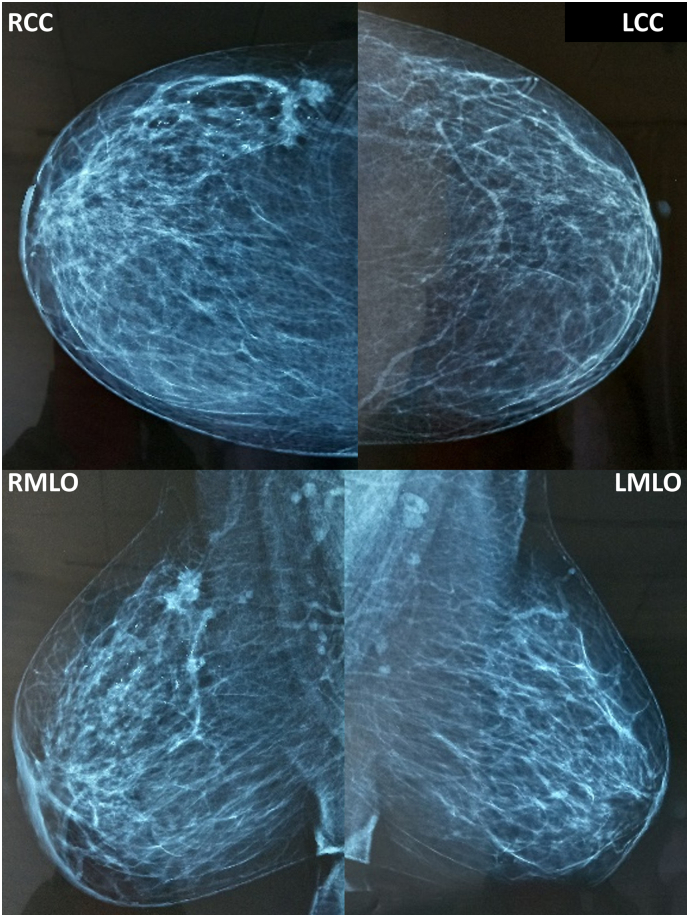
Patient 2- Mammogram showing spiculated densities in the upper outer quadrant of the right breast with parenchymal distortion, the largest measures 25 mm × 20 mm and multiple scattered microcalcifications were seen in the upper outer quadrant with multiple suspicious enlarged axillary lymph nodes. (RCC- Right cephalocaudal, LCC- Left cephalocaudal, RMLO- Right mediolateral oblique, LMLO- Left mediolateral oblique).

**Fig. 4:**
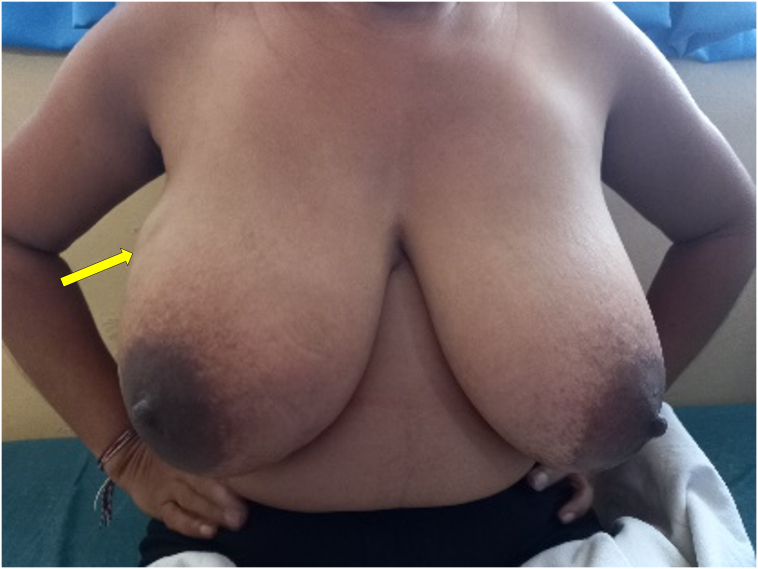
Patient 2 - Image at the time of presentation to the breast clinic showing fullness and a palpable lump in right breast.

A 39-year-old multiparous breastfeeding mother of a 2-year-old baby presented with a history of lumpiness in the right breast which she had noticed for 3–4 months. She has initially assumed the changes to be due to lactation. She sought medical attention after 3 months since the lump was not settling and was seen by a local general practitioner, who following examination had reassured her and managed the lump as a galactocele. However, she continued to seek medical attention as the lump was increasing and causing discomfort and distress. Throughout these medical visits she has been repeatedly reassured attributing the changes for lactation and imaging was not performed to assess the lump. She later presented to our breast clinic after a period of 6 months. Clinical assessment at our clinic revealed a 4 cm, firm lump in the right upper outer quadrant of the right breast with palpable axillary lymph nodes. She had no family history of breast cancer and had an uneventful past medical, surgical and allergy history. The mammogram showed spiculated densities in the upper outer quadrant of the right breast with parenchymal distortion, the largest measuring 25 mm × 20 mm. Multiple scattered microcalcifications were seen in the upper outer quadrant. Multiple suspicious enlarged axillary lymph nodes were noted with the largest measuring 22 mm × 20 mm. Ultrasonography revealed an inhomogeneous hypoechoic lesion with irregular margins measuring 24 mm × 22 mm suspicious of malignancy. Multiple enlarged lymph nodes were noted with loss of fatty hilum, largest measuring 20 mm in diameter (BIRADS IVB). Following her work up, she was diagnosed with an early stage invasive breast cancer, T2N1M0, Stage III. Her hormonal subtype was ER/PR+, HER2+ and Ki67 > 20 %. She underwent oncoplastic breast conserving surgery with lateral intercostal artery perforator flap reconstruction and axillary lymph node clearance by a senior consultant surgeon at a tertiary care centre and referred for adjuvant therapy (Fig. 3, Fig. 4).

### 2.3 Patient 3

**Fig. 5:**
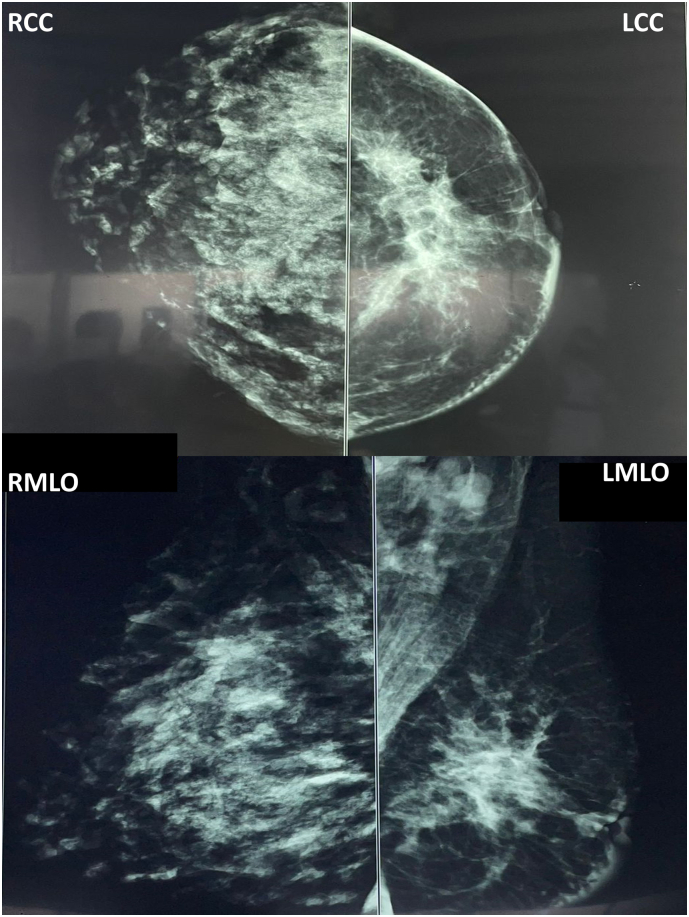
Patient 3 - Mammogram showing heterogeneously coarse, nodular and confluent prominent ductal patterns in the right breast compatible with lactation. There was an irregular shaped spiculated margined hyperdense lesion in the central region of the left breast associated with stromal distortion and nipple retraction. (RCC- Right cephalocaudal, LCC- Left cephalocaudal, RMLO- Right mediolateral oblique, LMLO- Left mediolateral oblique).

**Fig. 6:**
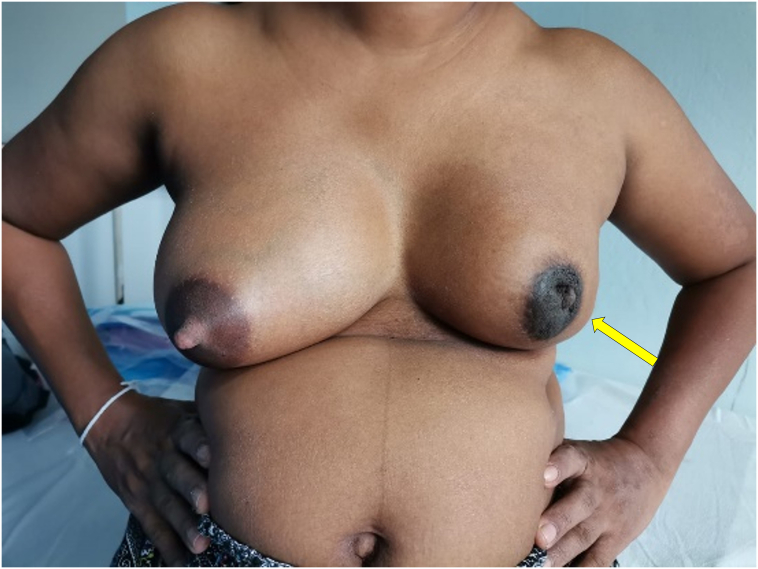
Patient 3 – Image at the time of presentation to the breast clinic showing significant breast asymmetry with nipple retraction.

A 38-year-old multiparous mother presented with left breast lump and discomfort for 5 months which she has noticed immediately following birth of her third child. She was seen and was treated as for edema-related early postpartum engorgement and galactocele during this course on several occasions. Initial ultrasound scan of breast report described the lesion as hypoechoic lobulated mass suggestive of lactating adenoma/galactocele and the BIRADS score was not mentioned. She presented to the breast clinic on her own due to the unresolving nature of the condition. Clinical assessment showed a significant asymmetry of the left breast with nipple retraction. She had no family history of breast cancer and had an uneventful past medical, surgical and allergy history. The mammogram revealed heterogeneously coarse, nodular and confluent prominent ductal patterns in the right breast compatible with lactation. There was an irregular shaped spiculated margined hyperdense lesion in the central region of the left breast measuring 65 mm × 49 mm × 34 mm in size associated with stromal distortion and nipple retraction. Skin thickening was also noted. Ultrasound scan showed an irregular shaped indistinct and angulated margined hypoechoic lesion in subareolar region measuring 37 mm × 19 mm in size. There was a prominent axillary lymph node with thickened cortex and altered fatty hilum measuring 9.5 mm in diameter (BIRADS V). Unfortunately, she was diagnosed with stage IV metastatic breast cancer with multiple bone metastasis (T3N2M1). Her hormone receptors were found positive for ER and PR, negative for HER 2 + ve and the Ki67 index of 10 %. She is currently undergoing palliative chemotherapy (Fig. 5, Fig. 6).

## 3 Discussion

Our case series represent very similar scenarios of delayed or missed diagnosis of breast cancer in young lactating women by both the primary care physicians as well as specialized medical practitioners. The most likely explanation is that breast cancer is an unlikely and uncommon presentation during lactation as opposed to breast edema, galactocele and lactation mastitis. Thus, there is less degree of clinical suspicion to consider breast cancer especially as it is uncommon in young women and even less during pregnancy and lactation.

Another plausible reason is that the clinical examination of a breast lump becomes quite challenging in the lactating breast. The mean breast weight doubles in pregnancy and lactation from 200 g to 400 g, resulting in increased firmness and density of the breast, which makes the interpretation of the clinical examination more difficult [4]. Thus, the sinister signs of malignancy are likely to be missed and that a diagnosis of breast cancer may not entertained early. Furthermore, all three patients had an ultrasonography of breast performed at least once and the radiological findings were attributed to physiological changes initially.

In depth evaluation of our case series revealed a number of red flag or sinister signs that cannot simply be attributed to lactation induced pathologies per se. The three mothers were nearing 40 years of age, making them come under the advanced maternal age group. Another interesting observation is that all three patients were having persistent non-resolving symptoms for >3–4 months requiring repeated medical attention. None of these patients had also developed typical features of lactation mastitis such as focal red area with fever or abscess formation. Also, prior aspiration was not performed in these patients to confirm a galactocele. Furthermore, the symptoms have not resolved with antibiotics which indicates a persisting underlying pathology. The three patients were also multiparous with no past history of problems in breast feeding. It is less likely for a multiparous mother to develop difficulty in establishing breast feeding causing breast edema leading to persistent mastitis. Furthermore, other red flags (at the time of presentation to the breast clinic) noted were firm or hard lump, dimpling of the skin, nipple changes, peau d'orange appearance and/or palpable painless axillary lymphadenopathy which were against the diagnosis of lactational mastitis.

Despite clinical assessment of the breast being difficult during pregnancy and lactation, a thorough examination especially of elderly mothers is safe and advisable. Particular attention should be paid to patients presenting with red flags and non-resolving symptoms. A triple assessment should be mandatorily carried out to avoid any missed diagnosis.

Ultrasonography is a sensitive (100 % sensitivity in one series) investigation method in pregnant and lactating women and is the preferred imaging modality because of the radio density of the breast in the pregnant or lactating state [11]. The most common sonographic finding is an irregular mass lesion with posterior acoustic enhancement. A marked cystic component is also seen occasionally. In our cohort, similar findings were observed such as irregular mass with cystic components.

Core needle biopsy and/or drainage may be an appropriate initial procedure with a sensitivity of 90 %; however, it may have slight increase in the risk of milk fistula formation, infection, and bleeding [12], [13]. A growing body of literature on postpartum breast cancer demonstrates poorer outcomes compared to other young women with breast cancer [14]. In some cases, delayed diagnosis plays a major role in these poor outcomes, while in others, it is more attributable to aggressive disease biology [14], [15].

In our country, strategies are needed to increase the awareness of breast cancer among the young females and also the practitioners, with regard to red flags of breast cancer among young females. A patient must be referred to a specialist center and a core biopsy of a lump should be performed in the context of persisting symptoms with red flags.

## 4 Conclusion

Lactational mastitis, breast abscesses, galactoceles, edema-related early postpartum engorgement are benign conditions that are unique in lactation period, but it is important not to overlook that the lactating women may develop any of the other breast problems seen in the non-lactating female population. Inadequate awareness of breast cancer among the young females and also the medical practitioners, with regard to red flags of breast cancer may be a reason for the delayed diagnosis in Sri Lanka. A lactating patient with persisting symptoms or red flags should be referred to a specialist center and/or a complete assessment of the breast should be performed to avoid missing a sinister diagnosis.

## Abbreviations

BIRADS: Breast Imaging Reporting and Database System score

## Consent

Written informed consent was obtained from the patients for publication of this case report and accompanying images. A copy of the written consent is available for review by the Editor-in-Chief of this journal on request.

## Ethics approval

Ethical approval is exempt/waived at our institution.

## Funding

N/A.

## Author contribution

Author KW, UJ, HG, SDS and ADS contributed to collection of information and writing of the manuscript. Authors KW, UJ and ADS contributed to the final approval of the manuscript. All authors read and approved the final version for publication.

## Guarantor

Dr. Kanchana Wijesinghe, Senior Lecturer and Consultant Surgeon, University Surgical Unit, Faculty of Medical Sciences, University of Sri Jayewardenepura, Sri Lanka.

## Research registration

Not applicable.

## Declaration of competing interest

N/A.
